# Dysfunction of histone demethylase IBM1 in *Arabidopsis* causes autoimmunity and reshapes the root microbiome

**DOI:** 10.1038/s41396-022-01297-6

**Published:** 2022-07-30

**Authors:** Suhui Lv, Yu Yang, Gang Yu, Li Peng, Shuai Zheng, Sunil Kumar Singh, Juan Ignacio Vílchez, Richa Kaushal, Hailing Zi, Dian Yi, Yuhua Wang, Shaofan Luo, Xiaoxuan Wu, Ziwei Zuo, Weichang Huang, Renyi Liu, Jiamu Du, Alberto P. Macho, Kai Tang, Huiming Zhang

**Affiliations:** 1 Shanghai Center for Plant Stress Biology, Center for Excellence in Molecular Plant Sciences, Chinese Academy of Sciences, Shanghai, 201602 China; 2 University of Chinese Academy of Sciences, Beijing, 100049 China; 3 Shanghai Key Laboratory of Plant Functional Genomics and Resources, Shanghai Chenshan Botanical Garden, Shanghai, 201602 China; 4 Haixia Institute of Science and Technology, Fujian Agriculture and Forestry University, Fuzhou, 350002 China; 5 Department of Biology, Southern University of Science and Technology, Shenzhen, 518055 China; 6 Department of Horticulture and Landscape Architecture, Purdue University, West Lafayette, IN USA; 7 Present Address: Instituto de Tecnologia Química e Biológica (ITQB), Oeiras, Lisbon, Portugal; 8 Present Address: Applied Sciences and Biotechnology, Shoolini University, Solan, 173212 India

**Keywords:** Plant sciences, Microbiology, Molecular biology

## Abstract

Root microbiota is important for plant growth and fitness. Little is known about whether and how the assembly of root microbiota may be controlled by epigenetic regulation, which is crucial for gene transcription and genome stability. Here we show that dysfunction of the histone demethylase IBM1 (INCREASE IN BONSAI METHYLATION 1) in *Arabidopsis thaliana* substantially reshaped the root microbiota, with the majority of the significant amplicon sequence variants (ASVs) being decreased. Transcriptome analyses of plants grown in soil and in sterile growth medium jointly disclosed salicylic acid (SA)-mediated autoimmunity and production of the defense metabolite camalexin in the *ibm1* mutants. Analyses of genome-wide histone modifications and DNA methylation highlighted epigenetic modifications permissive for transcription at several important defense regulators. Consistently, *ibm1* mutants showed increased resistance to the pathogen *Pseudomonas syringae* DC3000 with stronger immune responses. In addition, *ibm1* showed substantially impaired plant growth promotion in response to beneficial bacteria; the impairment was partially mimicked by exogenous application of SA to wild-type plants, and by a null mutation of *AGP19* that is important for cell expansion and that is repressed with DNA hypermethylation in *ibm1*. IBM1-dependent epigenetic regulation imposes strong and broad impacts on plant-microbe interactions and thereby shapes the assembly of root microbiota.

## Introduction

Plants are naturally inhabited by root microbiota, a variety of soil microbes surrounding or within the roots. While some soil microbes have no observable effects on plants, others can be either pathogens that cause detrimental effects on plants or beneficial species that promote plant growth and stress resistance [1, 2]. The complex community of root-associated microbes has been shown to be important for plant fitness [3–5], and is therefore emerging as an important target for soil management and for studying plant-microbe interactions [6–8].

Plant responses to microbes are controlled by an integrated network that consists of not only the immune system but also other intrinsic biological systems in planta [9–11]. Epigenetic features, such as DNA methylation and histone modifications, regulate gene transcription and genome stability through modulation of chromatin status [12]. Evidences are accumulating that epigenetic modifications can be involved in transcriptional regulation of plant disease resistance. For instance, DNA methylation in promoter regions limits transcriptional expression of *RMG1* and *RLP43*, two disease resistance genes in *Arabidopsis thaliana*, thereby negatively affecting plant resistance to the bacteria pathogen *Pseudomonas syringae* [13]. Although investigations have been reported about epigenetic regulation in plant disease resistance to individual pathogens, potential impacts of epigenetic regulation on the root microbiota compositions have been uncertain. In particular, while active DNA demethylation plays a positive role in plant resistance to pathogens such as *P. syringae* [13–17], defects in the RNA-directed DNA methylation (RdDM) pathway that establishes de novo DNA methylation caused elusive impacts on plant resistance to *P. syringae* [18–20]. In addition, genetic disruptions in the canonical RdDM pathway showed no alterations in *Arabidopsis* root microbiota [21], indicating that a role of plant epigenetic regulation in mediating aboveground responses to certain microbes does not necessarily assure an additional role in influencing the assembly of belowground root microbiota. Therefore, it remains an important question whether and how epigenetic regulation may affect the assembly of root-associated microbial communities.

Simultaneous alterations in DNA methylation and histone modifications can be more effective than single modifications or even necessary for epigenetic gene regulation [22, 23]. *Arabidopsis* IBM1 (Increased in Bonsai Methylation 1) is a histone H3 lysine9 (H3K9) demethylase whose dysfunction causes genome-wide increases in the levels of H3K9me2 and its allied DNA methylation in the CHG (H represents A, C, or T) context [24–27]. In this study, we investigated the root-associated microbiota in two *Arabidopsis* mutant alleles defective in IBM1. Microbiota profiling by 16S rRNA gene sequencing revealed that the *ibm1* mutations substantially reshaped the root-associated bacteria communities in the rhizosphere and the endosphere. Consistent with the altered plant-microbe interactions, IBM1 dysfunction leads to plant autoimmunity, as disclosed by the transcriptomic patterns including SA-mediated defense, systemic acquired resistance (SAR), and production of the defense metabolite camalexin. Analyses of genome-wide histone modifications and DNA methylation identified transcription-permissive epigenetic modifications at the loci of a group of key defense regulators, of which gene induction jointly contribute to the strong defense activation by IBM1 dysfunction. Compared to wild-type plants, *ibm1* showed increased resistance to the pathogen *P. syringae* with stronger immune responses to flg22. When exposed to a beneficial bacteria strain, *ibm1* showed impaired plant growth-promotion as a result of autoimmunity and the disruption in AGP19-dependent cell expansion. Therefore, IBM1-dependent epigenetic regulation is crucial for plant-microbe interactions including the assembly of root microbiota.

## Results

### IBM1 dysfunction reshapes root microbiota

To explore potential influence of IBM1-dependent epigenetic regulation on plant interactions with microbes, we performed 16S rRNA gene sequencing to investigate the root-associated (rhizosphere and endosphere) microbiota. Two *ibm1* mutant alleles, *ibm1-1* and *ibm1-4*, were compared with the wild-type Col-0 plants, in order to allow for stringent identification of IBM1-affected microbes by the overlap between the two mutant alleles. The plants were grown in natural soil substrates for 17 days under controlled environmental conditions.

A total of 2,041,913 effective tags were obtained with a median of 63,840 per sample (range 9384–146,838) across all the 35 samples. The effective tags were denoised and the resultant amplicon sequence variants (ASVs) were subjected to a cutoff with ≥5 reads in all the samples. Microbial species of the ASVs were annotated by using the QIIME2 software with the Silva138.1 database. After removing the ASVs that belong to Mitochondria, Chlorophyta, Archaea and Cyanobacteria, 4023 ASVs were obtained with a total of 1,629,593 feature counts and with a median of 46,011 per sample (range 6508–100,380). These ASVs were then normalized (see “Methods” for details) for subsequent analyses.

We first evaluated the effects of compartments on the microbiota assembly. The richness of ASVs in the three compartments were analyzed by the Chao1 estimator, which showed that the total numbers of ASVs, including either the observed ASVs or the estimated ASVs, were greater in the rhizosphere samples than the bulk soil and the endosphere samples (Fig. S1A–C). The higher ASV richness in the rhizosphere than the other two compartments likely reflects the fact that the rhizosphere is a nutrient-rich environment favorable for microbes. Meanwhile, the Shannon index analysis showed that the endosphere had a lower diversity of ASVs compared to those in the bulk soil and the rhizosphere (Fig. S1D), thereby reflecting the selectivity of plants on root-associated bacteria.

**Fig. 1:**
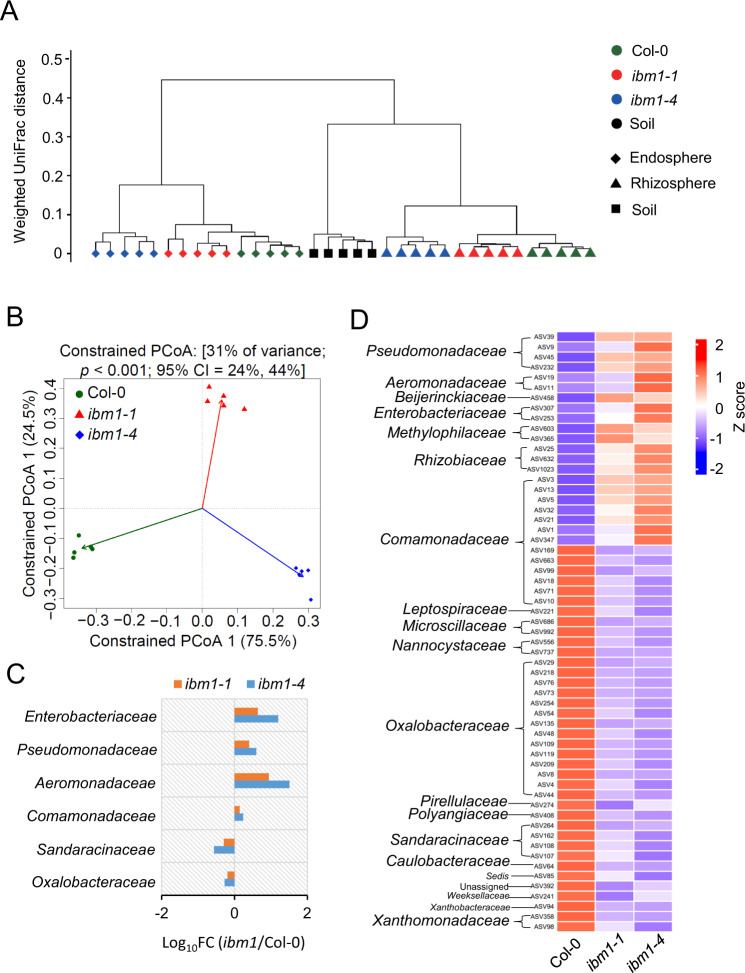
IBM1 dysfunction reshapes *Arabidopsis* root microbiome. **A** The hierarchical clustering of the weighted UniFrac distances between samples highlights the importance of compartment and genotype to microbiome. **B** The PCoA analysis of all ASVs separates the *ibm1* mutants from the wild-type plants (Col-0) within the endosphere compartment. **C** The *ibm1* mutations resulted in altered (FDR < 0.05 for both the *ibm1-1* vs Col-0 and the *ibm1-4* vs Col-0) RA in 6 bacteria families within the endosphere microbiome. The Log_10_ fold changes of mutant vs wild type are shown as horizontal bars. **D** The *ibm1* mutations resulted in altered (*p* < 0.05) RA in 59 endosphere ASVs, with 38 (64%) becoming less enriched in the mutants compared to the wild type. The notes on the left of the heatmap show the taxonomy of each ASV at the family level.

To compare the impacts of compartment and genotype on the bacteria community, we analyzed weighted UniFrac distances between all samples. The clustering patterns indicate that compartment is more influential than plant genotype in shaping the bacteria community (Fig. 1A); meanwhile samples were also clustered according to their genotype (Fig. 1A), indicating that the *ibm1* mutations altered root microbiota. Principal Coordinate Analysis (PCoA) of the weighted UniFrac distances showed that *ibm1-1* and *ibm1-4* were clearly separated from Col-0 in both the rhizosphere and the endosphere along the 1st coordinate, which accounted for 63.9% and 75.5% variations in the two compartments, respectively (Fig. 1B; Fig. S2A). Therefore, IBM1 plays an influential role in shaping root microbiota.

We profiled the taxonomic composition of the bacteria communities. At the level of phyla, *Proteobacteria* and *Bacteroidetes* displayed the highest and the second highest RA (Relative Abundance), respectively, in all three compartments (Fig. S2B; Dataset S1). *Bacteroidetes* accounted for approximately 14% of the ASVs in the bulk soil, while its RA was increased to more than 23% in the rhizosphere regardless of the plant genotype (Fig. S2B; Dataset S1), indicating that *Bacteroidetes* is generally more competitive than the minor phyla in the rhizosphere. By searching for microbiome alterations (FDR < 0.05) that were shared by the two *ibm1* mutant alleles, the phylum *Myxococcota* was identified as decreased in the endosphere, whereas the phyla of *Verrucomicrobiota* and *Myxococcota* were decreased in the rhizosphere (Dataset S1).

The *ibm1* mutations displayed strong impacts on the root microbiota at the level of families. Among the top 20 abundant families (Fig. S3), a total of 7 and 6 families showed altered RA (FDR < 0.05) in the rhizosphere and the endosphere, respectively, in both mutant alleles compared to Col-0 (Fig. 1C; Fig. S4A). In particular, 5 families showed *ibm1*-altered RA in both the rhizosphere and the endosphere, indicating that IBM1 is more influential on these bacteria families than the rest of the *ibm1*-affected families. These five families include *Oxalobacteraceae* and *Sandaracinaceae* which became less enriched, as well as *Pseudomonadaceae*, *Enterobacteriaceae*, and *Aeromonadaceae* which became more enriched in the *ibm1* mutants compared to Col-0. At the level of ASVs, the *ibm1* mutations commonly altered (*p* < 0.05) the RA of 59 ASVs in the endosphere, with 38 (64%) of the *ibm1*-affected ASVs becoming less enriched (Fig. 1D). Similarly, 76 ASVs in rhizosphere were identified as *ibm1*-affected, with 58 (76%) becoming less enriched in the mutants compared to the wild type (Fig. S4B). Altogether, these results demonstrate the influential role of IBM1 in the assemblage of root microbiota.

### IBM1 dysfunction activates defense responses in plant transcriptome

**Fig. 2:**
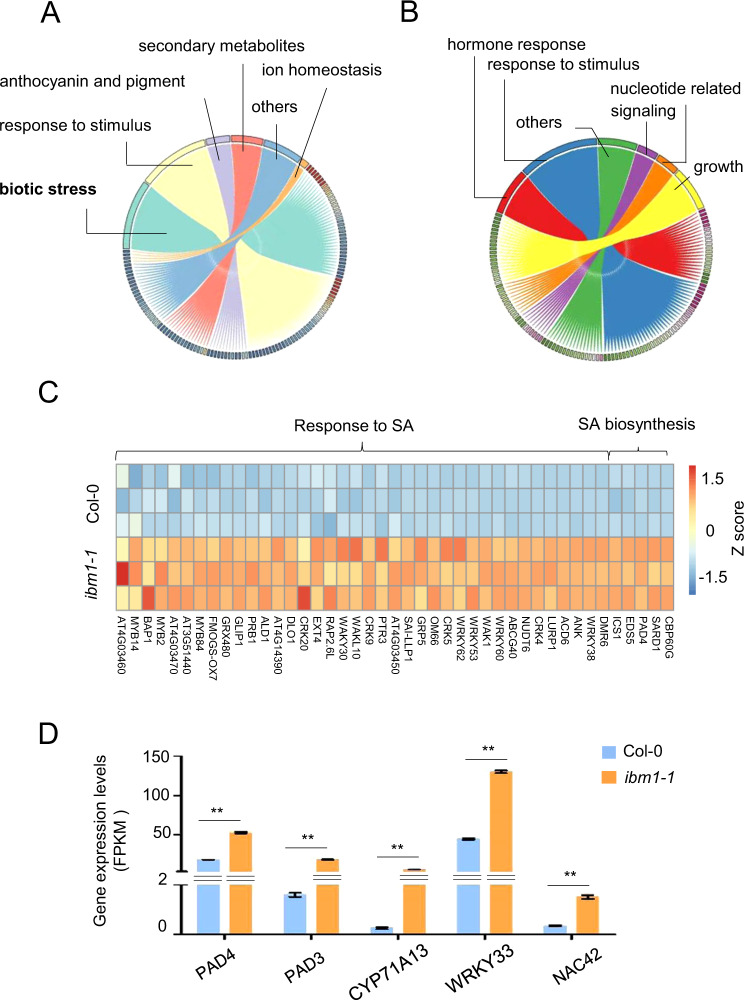
Transcriptome profiling disclosed plant autoimmunity caused by IBM1 dysfunction. The differentially expressed genes (DEGs; fold change ≥ 2, FDR ≤ 0.05) that were upregulated (**A**) and downregulated (**B**) in soil-grown *ibm1-1* compared to Col-0 were subject to the Gene Ontology (GO) analysis. The chord diagrams show the GO terms that link to their sub-classifications. The sub-classifications are labeled with GO ID that can be queried together with their corresponding DEGs in Dataset S2 (Sheets 3 and 5). **C** A heatmap of DEGs involved in SA signaling or biosynthesis. **D** Phytoalexin biosynthesis genes whose mRNA levels were increased by the *ibm1-1* mutation. Bars show the FPKM values from the RNA-seq results. Mean ± SE, *n* = 3 biological replicates. Double asterisks indicate statistical difference with *p* < 0.01 (Student’s *t*-test).

To understand the impacts on the root microbiota caused by IBM1 dysfunction, we performed RNA-seq to compare the transcriptome of soil-grown *ibm1-1* and Col-0 plants. In the 15-day-old plants, a total of 1396 and 498 differentially expressed genes (DEGs) were identified as upregulated and downregulated (fold change > 2, FDR < 0.05) by the *ibm1-1* mutation (Dataset S2). Gene ontology (GO) analysis highlighted GO enrichment of biotic stress-related process in the upregulated DEGs but not the downregulated DEGs (Fig. 2A, B; Dataset S2). This pattern indicated that dysfunction of IBM1 activates plant defense responses. Indeed, a group of 178 upregulated DEGs were categorized as defense, including 43 DEGs that were related to biosynthesis of or responses to salicylic acid (SA), a phytohormone critical for plant resistance to many pathogens (Fig. 2C; Fig. S5A; Dataset S2). Consistently, GO enrichment analysis revealed that IBM1 dysfunction activates systemic acquired resistance (SAR) (Fig. S5B), a plant immune response to pathogen attack. In addition, the upregulated DEGs showed enrichment (5/14, FDR = 0.023) of indole phytoalexin biosynthetic process (Fig. 2D; Dataset S2). Indole phytoalexins, including camalexin, are plant antimicrobial metabolites [28]. Thus, the transcriptional induction of phytoalexin biosynthesis indicated that IBM1 dysfunction activates both phytohormone-mediated and metabolite-mediated defense. The *ibm1-1* mutation upregulated a group of 16 genes involved in biosynthesis of flavonoids including anthocyanins (Fig. S5C; Dataset S2). This pattern implied stress responses in the *ibm1-1* plants, since elevated accumulation of flavonoids is a common indicator of plant stress responses [29]. These results collectively suggested that IBM1 dysfunction causes activated defense responses in the plant, thereby altering plant-microbe interactions and reshaping the root microbiota.

Because the plants were grown in non-sterile soil, it was possible that the defense responses in the *ibm1* mutant plants might result from microbial activation instead of directly from IBM1 dysfunction. To distinguish the two possibilities, we also performed RNA-seq to compare the transcriptomes of *ibm1-1* and Col-0 plants that were grown in sterile growth medium. A total of 1170 and 338 DEGs were identified as upregulated and downregulated (fold change > 2, FDR < 0.05) by the *ibm1-1* mutation (Dataset S2). Similar to the results of soil-grown plants, GO analysis highlighted GO enrichment of biotic stress-related processes in the upregulated DEGs but not the downregulated DEGs (Fig. S6A, B). The group of 206 upregulated defense DEGs include 43 DEGs that were related to biosynthesis of or responses to SA (Fig. S6C, D), as well as 18 DEGs that highlighted the activation of SAR (Fig. S6E). In addition, GO enrichment of upregulated DEGs involved in indole phytoalexin biosynthetic process (5/14, FDR = 0.019) and flavonoids biosynthesis (17/93, FDR = 0.000061) was also observed in the *ibm1-1* plants grown under the sterile conditions (Fig. S6F, G). Therefore, IBM1 dysfunction causes plant autoimmunity, since the transcriptional activation of defense responses in the *ibm1* mutants do not require external biotic stimuli.

### IBM1 dysfunction induces epigenetic changes permissive for transcription of key defense genes

**Fig. 3:**
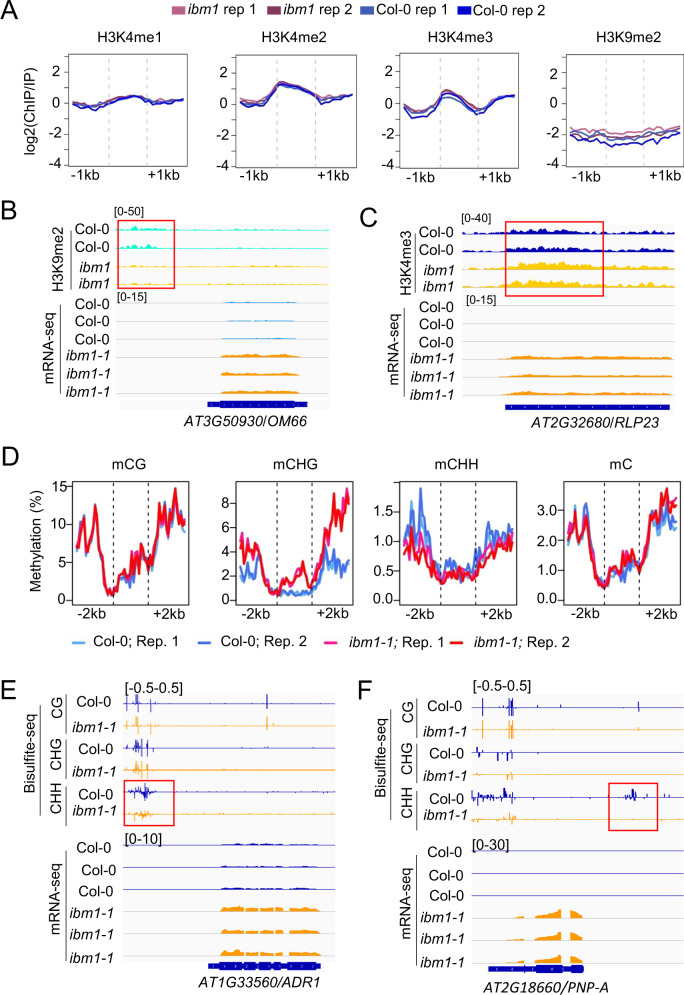
Epigenome analyses identified *ibm1* mutation-induced changes permissive for transcription of important defense genes. **A** The overall patterns of H3K4me1, H3K4me2, H3K4me3, and H3K9me2 levels at the 178 defense DEGs and the vicinity regions (up- and downstream of 1 kb). The original ChIP-seq data were downloaded from DDBJ (DRA005154) as generated previously (22). **B** IBM1 dysfunction decreases H3K9me2 level at the gene promoter of *OM66* and increases the mRNA level of *OM66*. Snapshots from ChIP-seq and RNA-seq are shown. The red box indicates the region with altered H3K9me2 levels. **C** IBM1 dysfunction increases H3K4me3 level at the gene body region of *RLP23* and increases the mRNA level of *RLP23*. **D** The overall patterns of DNA methylation levels at the 178 defense DEGs and the vicinity regions (up- and downstream of 2 kb). **E** IBM1 dysfunction decreases DNA methylation level at the promoter region of *ADR1* and increases the mRNA level of *ADR1*. Snapshots from whole-genome bisulfite sequencing and RNA-seq are shown. The red box indicates the gene promoter region with altered CHH methylation levels. **F** IBM1 dysfunction decreases DNA methylation level at the promoter region of *PNP-A* and increases the mRNA level of *PNP-A*.

Dysfunction of IBM1 causes transcriptional repression due to aberrant accumulation of H3K9me2, which can direct enzymatic removal of gene body H3K4me1, a histone modification for active transcription [24]. Given the molecular function of IBM1, we deduced that the transcriptional upregulation of the defense DEGs is a consequence of altered epigenetic features in the *ibm1* mutants. To investigate the potential epigenetic regulation of the defense DEGs, we analyzed the enrichment of H3K9me2, H3K4me1, H3K4me2, and H3K4me3 at the 178 defense DEGs by using a previously reported *ibm1* ChIP-seq (chromatin immunoprecipitation-sequencing) dataset (DDBJ: DRA005154) [24]. None of the examined histone marks showed an overall correlation with all the defense DEGs (Fig. 3A). However, changes in single or multiple histone modifications were observed at 32 individual loci of the defense DEGs, including their upstream and downstream 1 kb regions (Table S2). These loci include 9 and 9 DEGs with increased and decreased H3K9me2, respectively; meanwhile, increased and decreased levels of H3K4me1/2/3 were observed at 14 and 3 defense DEGs, respectively (Fig. S7; Table S2). Importantly, the level of the repressive H3K9me2 was decreased by IBM1 dysfunction at the promoter of *OM66* (*Outer Mitochondrial membrane protein of 66* *kDa*) (Fig. 3B), of which overexpression causes constitutive induction of SA-dependent defense gene expression and increased resistance to *P. syringae* [30]. IBM1 dysfunction also causes decreased H3K9me2 in the promoter region of *SIB1* (*SIGMA FACTOR BINDING PROTEIN 1*) (Fig. S7A), a plant defense regulator whose overexpression leads to enhanced resistance to *P. syringae* and the necrotrophic pathogen *Botrytis cinerea* [31, 32]. In addition, the level of the permissive H3K4me3 was increased by IBM1 dysfunction within the gene body region of *RLP23* (*Receptor-like protein 23*) (Fig. 3C), which mediates plant immune activation in response to multiple microbial species [33]. Increased levels of gene body H3K4me3 were also induced by IBM1 dysfunction at the loci of *DHYPRP1* (*DOUBLE HYBRID PROLINE-RICH PROTEIN 1*) and *CRK45* (*Cysteine-rich Receptor-like protein Kinase 45*) (Fig. S7B). Overexpression of CRK45 in *Arabidopsis* increased expression of defense genes and enhanced resistance to *P. syringae* [34], while AtDHYPRP1 overexpressing lines exhibited enhanced resistance to *P. syringae* [35]. These findings demonstrate that IBM dysfunction leads to permissive chromatin environments for transcription of multiple important defense genes, thereby at least partially explaining the altered immune responses and root microbiota in the *ibm1* mutants.

To investigate whether DNA methylation plays a role in the upregulation of defense DEGs, we performed whole-genome bisulfite sequencing to investigate *ibm1* methylome by using the same soil-grown plant samples as for RNA-seq. Dysfunction of IBM1 results in 14621 hyper DMRs (differentially methylated regions) and 853 hypo DMRs (Dataset S3). The overwhelming majority of hyper DMRs were CHG hypermethylation that locates in protein-coding gene regions (Fig. S8A). In contrast, the hypo DMRs were mostly contributed by CHH hypomethylation, which showed a preference to promoter regions (38.1%) compared to the other types of regions, including intergenic regions (25.1%), protein-coding regions (17.9%), and transposable elements (18.9%) (Fig. S8B).

At the 178 defense DEG loci, the average levels of CHG methylation and CHH methylation were increased and decreased, respectively, in *ibm1-1* compared to Col-0 (Fig. 3D). While CHG cytosines generally show higher methylation levels than CHH cytosines, these defense DEG loci harbor more CHH cytosine than CHG cytosine; as a result, the average levels of total cytosine methylation were similar between *ibm1-1* and Col-0 at these loci(Fig. 3D). Although the transcriptional upregulation of the defense DEGs did not show an overall correlation with total DNA methylation levels, it appeared to be positively correlated with CHG hypermethylation and/or CHH hypomethylation. Examinations of individual locus revealed CHH hypomethylation at the promoter regions of 23 defense DEGs loci (Table S2). Particularly, IBM1 dysfunction caused DNA hypomethylation in the promoters of the upregulated DEGs including *WAKL10* (wall-associated kinase-Like 10), *PNP-A* (Plant Natriuretic Peptide A), *ADR1* (Activated Disease Resistance 1), and *RMG1* (Resistance Methylated Gene 1) (Fig. 3E, F; Fig. S8C, D). The *Arabidopsis* null mutant *wakl10* plants exhibited increased susceptibility to *P. syringae* [36]; while overexpression of PNP-A in *Arabidopsis* increased expression of defense genes and enhanced resistance to *P. syringae* [37]. Overexpression of ADR1 in *Arabidopsis* resulted in constitutive activation of SA-dependent defense responses and increased resistance against several biotrophic pathogens [38]. In particular, DNA hypomethylation in the promoter region of *RMG1* has been shown to promote its gene expression and plant resistance to *P. syringae* [13]. Therefore, the transcriptional activation of defense responses by IBM1 dysfunction can be at least partially attributed to direct epigenetic regulation. Gene induction of these four DEGs, together with *OM66* and the other defense DEGs associated permissive histone modifications, collectively demonstrate a strong impact of IBM1 dysfunction on plant immune responses.

### IBM1 dysfunction causes opposite impacts on plant responses to pathogenic and beneficial bacteria

**Fig. 4:**
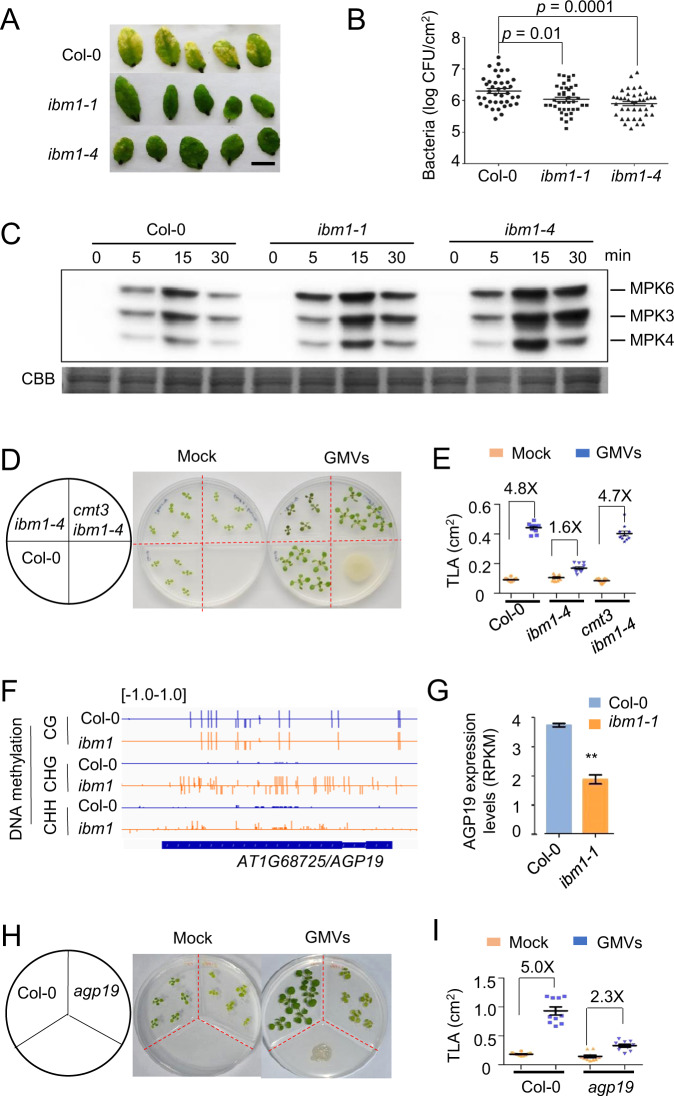
IBM1 dysfunction increases plant disease resistance and impairs growth promotion triggered by beneficial bacteria. **A** The disease resistance phenotype of *ibm1-1*, *ibm1-4*, and Col-0. Five-week-old plants were injection-inoculated with *Pst*. DC3000 (10^5^ cfu/mL). The leaves shown were photographed at 5 dpi (days post inoculation). Scale bar = 1 cm. **B** Bacterial growth of *Pst*. DC3000 on plants at 3 dpi. Mean ± SE, *n* = 40 individual plants. Student’s *t*-test *p* values are shown. **C** Flg22-induced MPK3/MPK6 phosphorylation is enhanced by IBM1 dysfunction. Kinase assays were performed with samples harvested at 0, 5, 15, and 30 min after the flg22 treatments. Two independent experiments were performed with similar results. **D** IBM1 dysfunction impairs plant growth promotion triggered by GB03-produced microbial volatiles (GMVs); the impairment can be rescued by a second mutation of *cmt3* in the *ibm1* mutant. Images were taken at 7 days after treatment (DAT). Red-dotted lines indicate inner plastic partitions that divide the plate into four parts. **E** Quantification of total leaf area per seedling (TLA) of the plants at 7 DAT. Mean ± SE, *n* = 15. All fold changes are associated with statistical significance of *p* < 0.01 (Student’s *t*-test). **F** IBM1 dysfunction increases DNA methylation levels in the CHG and CHH contexts at the *AGP19* locus. A snapshot from whole-genome bisulfite sequencing is shown. **G** IBM1 dysfunction decreases the mRNA level of *AGP19*. Bars show the FPKM values from the RNA-seq results. Mean ± SE, *n* = 3 biological replicates. Double asterisks indicate statistical difference with *p* < 0.01 (Student’s *t*-test). **H** The *agp19* null mutant showed impaired plant growth promotion by GMVs. Images were taken at 7 days after treatment (DAT). **I** Quantification of total leaf area per seedling (TLA) of the plants at 7 DAT. Mean ± SE, *n* = 15. All fold changes are associated with statistical significance of *p* < 0.01 (Student’s *t*-test).

To examine the impacts of IBM1 dysfunction on plant disease resistance, we compared the *ibm1* mutants with wild type plants in response to *P. syringae* pv. *tomato* DC3000 (*Pst*. DC3000). Consistent with the transcriptional activation of defense responses, the *ibm1* mutants showed increased disease resistance, as indicated by the lower levels of pathogen infection and the resultant disease symptom (Fig. 4A, B). The increased disease resistance was further supported by the enhanced activation of MPK3 and MPK6, which are two immune-related MAP kinases critical for robustness of the immune signaling network [39, 40], in the *ibm1* mutants challenged by the bacterial flagellin epitope flg22, a pathogen-associated molecular pattern (PAMP) that elicits PAMP-triggered immunity (PTI) (Fig. 4C). Consistently, flg22 induced higher levels of the PTI marker gene *FRK1* in the *ibm1* mutants than the wild type plants (Fig. S9A). Compared to wild-type plants, the *ibm1* mutants showed lower levels of reactive oxygen species (ROS) in response to flg22 (Fig. S9B), indicating that ROS burst is not a key factor for the increased disease resistance in *ibm1*. Given that IBM1 dysfunction causes activation of SA-mediated defense responses and the enhancement of plant resistance to *Pst*. DC3000, it appears that the *ibm1* mutants resemble the *Arabidopsis sik1* mutants, which also showed decreased ROS levels in response to flg22 but enhanced resistance to *Pst*. DC3000 due to strong activation of SA-mediated defense [41].

To further investigate the impacts of IBM1 dysfunction on plant-microbe interactions, plants were exposed to *Bacillus amyloliquefaciens* GB03, a plant-beneficial bacterium capable of triggering plant growth promotion through volatile emissions [42]. IBM1 dysfunction strongly impaired plant growth promotion triggered by GB03-produced microbial volatiles (GMVs), whereas a second mutation in either the H3K9 methylase *KYP/SUVH4* or the CHG DNA methylase *CMT3* restored the plant growth promotion (Fig. 4D, E; Fig. S10A, B), further demonstrating the importance of epigenetic regulation on plant-microbe interactions. Suppression of the GMV-triggered plant growth-promotion was mimicked by exogenous applications of SA and JA (Fig. S10C, D), supporting a role of the activated defense responses by IBM1 dysfunction in antagonizing plant growth-promotion.

IBM1 dysfunction caused CHG and CHH hypermethylation across the locus of *AGP19* (Arabinogalactan protein 19), concomitant with downregulated gene expression of *AGP19* (Fig. 4F, G). Dysfunction of AGP19 resulted in pleiotropic phenotype including abnormal cell expansion [43], whereas GMV-triggered plant growth-promotion involves leaf cell expansion [42]. Thus, we examined a null T-DNA knockout mutant of AtAGP19. Compared to the wild-type plants, *agp19* showed substantially reduced plant growth promotion in response to GMVs (Fig. 4H, I). Therefore, in addition to epigenetic activation of the defense DEGs, epigenetic suppression of AGP19 likely also contributes to the altered plant-microbe interactions in the *ibm1* mutants.

## Discussion

Important roles of epigenetic regulation have been implicated in plant-microbe interactions and the mechanisms are becoming increasingly clear [13, 44, 45]. Nonetheless, little is known about whether and how the assembly of plant-associated microbiota may be controlled by epigenetic regulation. Our findings in this study demonstrate a crucial role of IBM1-dependent epigenetic regulation in shaping root microbiota. In addition, our results of mechanistic investigations highlight a combination of key defense regulators, of which gene induction can be attributed to or correlated with direct epigenetic regulation, resulting in alterations in plant interactions with both pathogenic and beneficial bacteria.

CHH hypomethylation was observed at the promoter regions of several key defense regulators such as *ADR1* and *RMG1*, which concomitantly showed gene upregulation in the *ibm1* mutants. DNA methylation in the asymmetric CHH context is established through the RdDM pathway [12]. Because *A. thaliana* mutants with defects in the canonical RdDM pathway showed similar root microbiota as the wild-type plants [21], we wondered whether defects in the RdDM pathway would also lead to transcriptional upregulation of those defense regulators. Quantitative RT-PCR measurements showed that, among those nine defense regulators (WAKL10, OM66, RLP23, ADR1, CRK45, SIB1, RMG1, DHYPRP1, and PNP-A), eight genes showed no increased expression in the *Arabidopsis nrpd1-3* or *nrpe1-11* mutants (Fig. S11A), which are defective in RNA polymerase IV (Pol IV) and Pol V, respectively, two core components of the canonical RdDM pathway; meanwhile DHYPRP1 showed increased gene expression in *ibm1-1* and *nrpe1-11*, but not *nrpd1-3* (Fig. S11A). In addition, unlike *ibm1* mutants, the *nrpd1-3* and *nrpe1-11* mutants showed similar plant growth promotion as wild-type plants in response to GMVs (Fig. S11B, C). Simultaneous alterations in DNA methylation and histone modifications can be more effective than single modifications or even necessary for epigenetic gene regulation [22, 23]. Because the defects in RdDM are not sufficient to cause gene induction of the defense regulators, it is likely that epigenetic upregulation of those genes additionally (or solely) requires changes in certain histone marks, which may be present in the *ibm1* mutants but not the RdDM mutants. Nonetheless, the differential regulation of these key defense genes demonstrates that IBM1 has stronger impacts on plant defense than Pol IV and Pol V, and that the strong impacts on plant-microbe interactions by IBM1 dysfunction should be attributed to a combinational outcome of gene induction of the defense regulators.

SA-mediated plant defense was activated in the *ibm1* mutants (Fig. 2; Figs. S5, S6), meanwhile the enrichment of *Pseudomonadaceae* and *Oxalobacteraceae* was increased and decreased, respectively, in both the rhizosphere and the endosphere in the *ibm1* mutants compared to wild type plants (Fig. 1). SA-dependent immune signaling modulates root colonization by specific bacteria families [46]. In particular, increased microbiota enrichment of *Pseudomonadaceae* was observed in the *cpr1* and *cpr5* mutants, both of which constitutively activate SA-dependent defense signaling; whereas decreased enrichment of *Oxalobacteraceae* was observed in the mutant *pad4* that has defective SA signaling [46]. Therefore, it is likely that the activated SA signaling is causal to the modulation of *Pseudomonadaceae* and *Oxalobacteraceae* in the *ibm1* mutants. In addition, IBM1 dysfunction transcriptionally upregulates a group of genes responsible for the production of camalexin (Fig. 2D; Fig. S6G), which is a major defense metabolite that can be secreted to inhibit pathogen growth [47]. Moreover, the downregulation of *AGP19* in *ibm1* mutants also contributes to the interrupted interactions between the plant and the beneficial bacteria. Thus, IBM1-dependent epigenetic regulation plays a crucial role in mediating multiple mechanisms underlying plant-microbe interactions.

In this study, we found that *IBM1* dysfunction enhanced plant resistance to the pathogen *P. syringae*. This phenotype is consistent with the *ibm1* transcriptome that showed autoimmunity in both the soil-grown plants and the plants grown in sterilized medium. Particularly, the enhanced disease resistance is consistent with the *ibm1* mutation-induced upregulation of the defense-related genes such as *OM66*, *PNP-A*, and *RMG1*, whose elevated gene expression has been shown to increase plant resistance to *P. syringae* [13, 30, 37]. In addition, the flg22-treated *ibm1* mutants showed stronger activation of MPK3/4/6 and stronger induction of *FRK1* compared to the wild-type plants. These enhanced PTI responses also support the enhanced plant disease resistance. Because *IBM1* dysfunction causes defects in the production of stomatal lineage cells [48], our study used the leaf-infiltration method for the *P. syringae* treatments. We observed that the *ibm1* mutants, compared to the wild-type plants, had less intensive yellow color (i.e., chlorosis) that is typical to *P. syringae*-triggered disease. Intriguingly, the *ibm1* mutants treated with *P. syringae* by leaf-dipping were reported to show decreased disease resistance, as shown by the more intensive brownish color on the *ibm1* leaves than the wild-type leaves [49]. While the discrepancy between our study and Chan et al. [49] may result from different experimental systems, the different observations suggest that the impacts of *IBM1* dysfunction on plant defense can be influenced by certain unidentified factors.

Genome-wide disturbance of epigenetic patterns can result in pleiotropic morphological phenotypes that accumulate during propagation of the mutants, such as *ddm1*, *met1*, and *ibm1* [26, 50, 51]. Plant interactions with the root microbiome are affected by not only in planta immunity but also the root morphology and root exudates. In addition, mutations in IBM1 cause developmental defects in stomata [48]. If this affects gas exchange for photosynthesis, it probably would cause changes in the quantity/composition of the photosynthates secreted from the root, thereby contributing to the changes in the root microbiome. Therefore, it is not surprising to see variations in root microbiome compositions between different *ibm1* mutant alleles; meanwhile, the comparison between different alleles would help increase the confidence in identifying the bacteria community members that are truly affected by IBM1, since they are commonly present in the two different mutant alleles.

The dysfunction of IBM1 results in genome-wide disruptions in the methylome, the transcriptome, and consequently the pleiotropic phenotype including the elevated plant defense. Our attempts in finding a master regulator that is directly targeted by IBM1 did not succeed; instead, we identified a group of DEGs that are known to be important for plant defense and that showed epigenetic alterations in the *ibm1* mutants. We deduced that the transcriptional alterations in this group of defense-related genes jointly mediate, at least partially, the elevated defense in the *ibm1* mutants. In addition, genetic disruptions in the canonical RdDM pathway did not mimic IBM1 dysfunction in causing either the transcriptional regulation of these defense-related genes or the impacts on the plant-microbe interactions, indicating that the defense-related impacts observed in *ibm1* are not common outcomes from epigenomic disruptions. While all the impacts on plant-microbe interactions in *ibm1* certainly originated from a loss of the IBM1-dependent epigenetic regulation, it remains unclear how the immediate impacts of IBM1 dysfunction eventually leads to the pleiotropic effects.

## Conclusions

The histone demethylase IBM1 in *A. thaliana* has strong influences on the assembly of root microbiota, as explained by its essential role in preventing SA-mediated autoimmunity. In particular, dysfunction of IBM1 leads to epigenetic modifications that are permissive for gene transcription at the loci of a group of important defense regulators. Concomitantly, dysfunction of IBM1 displayed strong impacts on plant binary relations with either pathogenic or beneficial bacteria. Our comprehensive analyses demonstrate the strong and broad impacts of epigenetic regulation on plant–microbe interactions particularly the assembly of root microbiota.

## Methods

### Plant materials and growth conditions

Seeds were stratified at 4 °C for 2 days before sowed. After surface sterilized with 30% household bleach for 15 min and washed with ddH_2_O at least five times, seeds were dispersed on half-strength Murashige and Skoog (MS) medium containing 0.7% agar and 1% sucrose and placed in the growth chamber. The growth chamber is set with a 16 h light/8 h dark cycle with a total light intensity of 120 µmol m^−2^ s^−1^, a temperature of 22 °C and a relative humidity of 60%.

All plants used in this study were in *Arabidopsis* Col-0 background. The mutants of *ibm1-1* (point mutation) and *ibm1-4* (SALK _035608) were described previously [26]. The double mutants of *cmt3-11t ibm1-4* and *ibm1-4 kyp* were generated previously [48]. The single mutants of *cmt3-11t* (SALK _148381), *kyp* (SALK _044606), *nrpd1-3* (SALK _128428) and *nrpe1-11* (SALK _029919) were from Prof. Jian-Kang Zhu’s lab at Shanghai Center for Plant Stress Biology. The mutant *agp19* (SALK _038728) was ordered from Nottingham Arabidopsis Stock Center.

### Sample preparation and library construction for 16S rRNA gene sequencing

Natural soil was collected from Chenshan Botanical Garden at Shanghai, China. The soil was homogenized and mixed with commercial soil (Pindstrup Substrate) with 2:1 ratio, and placed into 10 × 10 × 8 cm pots. The 14-day-old seedlings of Col-0, *ibm1-1* and *ibm1-4* were transplanted from sterile growth medium into the pots (five seedlings for each pot), and were grown for another 17 days in a growth room at 22 °C under 16 h light/8 h dark condition. Five biological replicates were prepared for each genotype and each biological replicate contains 10 plants. Unplanted pots were subjected to the same conditions as the planted pots to prepare the control soil samples at harvest.

The rhizosphere samples and root endophyte samples were harvested according to Schlaeppi et al. [52]. DNA were extracted by using FastDNA SPIN kit for Soil (MP Biomedicals, Solon, USA) with minor modifications. Samples were homogenized in the Lysis Matrix E tubes using a Retsch MM400 mill at a frequency of 30 Hz for 30 s. After extraction, DNA samples were eluted in 50–100 µL DES water and DNA concentrations were determined using the Qubit dsDNA HS Assay Kit (Invitrogen, Life Technologies) on Qubit2.0 (Life Technologies, USA).

The 16S rRNA gene amplicon generation and library preparation were performed as described previously [53]. Amplicon libraries were generated using the PCR primers 799F (AACMGGATTAGATACCCKG) and 1193R (5′-ACGTCATCCCCACCTTCC-3′) [54, 55]. The first amplification was performed in a 25 µL reaction volume, including 2.5 µL microbial DNA (5 ng/µL), 5 µL forward primer (work concentration 1 µM), 5 µL reverse primer (work concentration 1 µM), 12.5 µL 2X KAPA HiFi Hot Start Ready Mix. The PCR setting was 95 °C for 3 min, 30 cycles of 95 °C for 30 s, 55 °C for 30 s, 72 °C for 30 s, and 72 °C for 5 min. For each biological replicate, three technical replicates were used. The second amplification was conducted in 20 µL reaction volume, each containing 10 µL 2 X KAPA HiFi Hot Start Ready Mix, 200 nM 2P-F and 2P-R primer, 2 nM F-(N) and R-(N) primer, and 40 ng first-round PCR product. For each biological replicate, two technical replicates were determined. The PCR conditions were 95 °C for 3 min, 10 cycles of 95 °C for 30 seconds, 55 °C for 30 s, 72 °C for 30 s, and 72 °C for 2 min. All the primers sequences were included in Table S1. After each PCR, the PCR products were loaded on 2% agarose gel and cut from the gel with Tanon UV-2000 gel imaging system and extracted from the agarose using the QIAquick Gel Extraction kit (QIAGEN). DNA concentrations were determined using the Qubit dsDNA HS Assay Kit (Invitrogen) on Qubit2.0 (Life Technologies, USA).

After the two rounds of PCR, five biological replicates for each genotype were combined, and DNA concentrations were determined using the Qubit dsDNA HS Assay Kit (Invitrogen, Life Technologies) on Qubit2.0 (Life Technologies, USA). Then all the genotype samples were combined with the same amount of each genotype PCR product.

### 16S rRNA gene sequencing and data analysis

The libraries were sent to the Novogene company for 16S rRNA gene sequencing by paired-end sequencing based on a Hiseq2500-PE250 platform (Illumina). Processing and statistical analysis of 16S rRNA gene counts was performed by the Novogene Company and the Core Facility of Bioinformatics in Shanghai Center for Plant Stress Biology, China.

Paired-end reads were assigned to samples based on their unique barcodes and were truncated by cutting off the barcodes and primer sequences. Paired-end reads were merged using FLASH v1.2.11 [56]. Quality filtering on the raw tags were performed using the fastp software to obtain high-quality Clean Tags, which were compared with the reference database Silva138.1 using Vsearch to detect the chimera sequences, and then the chimera sequences were removed to obtain the Effective Tags [57]. Denoise was performed with DADA2 module in the QIIME2 software to obtain initial amplicon sequence variants (ASVs). ASVs with total abundance less than 5 were filtered out. Species annotation was performed by using the QIIME2 software based on Silva138.1 database. The ASVs which belongs to Mitochondria, Chlorophyta, Archaea and Cyanobacteria were removed. The minimum number of reads in the sequencing samples was selected as the base, and the number of reads in all samples was uniformly extracted to this value for normalization of differences in sequence depth. The analyses of alpha diversity and beta diversity were performed by using QIIME2 based on the normalized data. All figures were produced by in-house R program. The relative abundance of ASVs was calculated by dividing the reads per ASV in a sample by the sum of the usable reads in that sample. To find out the significantly different species at phylum and family levels, the R software was used to do the MetaStat analysis. The resulting *p* values for phylum and family comparisons were adjusted by Benjamini-Hochberg false discovery rate (FDR) correction for multiple hypotheses testing.

### mRNA-seq and data analysis

For RNA-seq of plants grown in soil, 5-day-old seedlings of Col-0 and *ibm1-1* were transferred from 1/2 MS plate into the soil, and grown for another 10 days before whole seedlings were harvested for RNA extraction. Each sample included three biological replicates with 15 seedlings for each replicate. For RNA-seq of plants grown in sterile medium, 7-day-old seedlings of Col-0 and *ibm1-1* grown in 1/2 MS medium were collected for RNA extraction. Each sample had two biological replicates. Total RNA was extracted from the samples by RNeasy Plant Mini Kit (QIAGEN). Library construction and deep sequencing were performed by the Core Facility of Genomics in Shanghai Center for Plant Stress Biology, China. An aliquot with 1 μg total RNA per sample was used for library preparation with the NEBNext Ultra Directional RNA Library Prep Kit (New England Biolabs; E7420L) for Illumina.

The raw reads of RNA-seq of soil-grown plants were processed by SolexaQA [58] and cutadapt [59] to remove low-quality reads and adapter sequences. Then the reads, with the length of more than 25 bp and phred score of greater than 17, were mapped to the *Arabidopsis* TAIR10 genome using TopHat2.0.10 [60] with default. The reads mapped to each annotated gene were counted by HTseqcount [61]. The raw counts of each gene were normalized using edgeR [62], and differentially expressed genes were identified using fold change >2 and false discovery rate <0.05 as significance cutoffs. The paired-end reads of medium RNA-seq were cleaned by Trimmomatic (version 0.36) [63]. After trimming the adapter sequence, removing low-quality bases and filtering short reads, clear read pairs were retained for further analysis. The cleaned reads were mapped to the *A. thaliana* reference genome sequence downloaded from TAIR10 by HISAT with default parameters [64]. Number of reads that were mapped to each gene was calculated with the *htseq-count* script in HTSeq [61]. The heatmap was prepared using R statistics package.

### Quantitative real-time PCR

RNA was extracted by RNeasy Plant Mini Kit. For mRNA expression analysis, total RNA was used for reverse-transcription by oligo (dT)_18_ from EasyScript® One-Step gDNA Removal and cDNA Synthesis SuperMix (Trans, AE311-02) according to its manufacturer’s instructions. Real-time PCR was carried out using iQ SYBR Green Supermix (Bio-Rad) on a CFX96 real-time PCR detection system (Bio-Rad). The housekeeping gene, *ACTIN2*, was used as the internal control for all reactions. Relative gene expression was derived by using 2^−ΔΔCT^, where ΔCT represents CT of the target gene minus CT of the reference gene *ACTIN2*. All primers used are in Table S3.

### Whole-genome bisulfite sequencing and data analysis

For whole-genome bisulfite sequencing, 5-day-old seedlings of Col-0 and *ibm1-1* were transferred from 1/2 MS plate into the soil, and grown for another 10 days before the whole seedlings were harvested for DNA extraction using DNeasy Plant mini kit (QIAGEN; Cat. No. 69104). Two biological replicates for each sample were prepared with 15 seedlings for each replicate. Bisulfite conversion, library construction and deep sequencing were performed by the Core Facility of Genomics in Shanghai Center for Plant Stress Biology, China. An aliquot with 1 μg DNA per sample was used for library preparation with EpiTect Plus DNA Bisulfite Kit (QIAGEN; Cat. No. 59124) and the NEBNext Ultra II DNA Library Prep Kit (New England Biolabs; E7645L) for Illumina. The libraries were sequenced on the HiSeq2500 (Illumina).

The raw reads were mapped to the naturally unmethylated chloroplast genome of *Arabidopsis* using Bismark_v0.19.0 in order to evaluate the bisulfite non-conversion rate. The libraries with non-conversion rates of <1% were retained for further analysis. Adapters and low-quality reads were trimmed using cutadapt. Then the reads were uniquely mapped to each corrected pseudo-reference genome using Bismark_v0.19.0 (bismark -bowtie2 -X 1000 -N 1). After filtering the duplicate reads, the methylation information for each cytosine site was extracted and only sites with at least four mapped reads covered were considered. In the binomial test, the non-conversion rate was used as the expected probability.

Differentially methylated regions (DMRs) were identified using methylPipe [65]. To avoid the comparison between two DMRs both with low methylation level, we required the difference of mean methylation level between mutant and WT to be ≥0.1, with a fold change ≥2 and a *p* value of less than 0.05. Differentially methylated cytosines (DMCs) were identified using metilene, and DMRs were further filtered to contain at least ten DMCs, and each DMC is with a fold change ≥2, a difference of methylation rates larger than 10% and a P value of less than 0.05.

### ChIP-seq data analysis

The public H3K4me1/2/3 and H3K9me2 ChIP-seq data were downloaded from DDBJ with the accession number DRA005154 [24]. The downloaded single-read data were trimmed using Trimmomatic [63] with parameters “LEADING:20 TRAILING:20 SLIDINGWINDOW:4:15 MINLEN:30”. The trimmed reads were mapped to TAIR10 genome using tool Bowtie [66] with parameters “-m 1 -v 0”. To remove potential PCR duplicates, the “rmdup” command of SAMtools [67] was used.

### Plant growth-promotion assay

For GB03 VOCs treatment, according to Zhang et al. [42], 5-day-old seedlings were transferred into the Petri dish with three-divided partitions. PGPR strain *Bacillus subtilis* GB03 stock was cultured in liquid LB at 37 °C overnight, one day before plant experiments. To the none-plant partition of the Petri dish, 20 µL of GB03 suspension culture was applied, thus plants are exposed to bacterial VOCs without physical contact. The Petri dishes were sealed with parafilm. The total leaf area per seedling was measured by Image J.

### Measurements of reactive oxygen species

Reactive oxygen species (ROS) measurements were performed in *Arabidopsis* plants as described previously [68]. Well-expanded rosette leaves of 4-week-old health plants, grown in the short-day condition (22 °C, 10 h light/14 h dark, 120 µmol photons m^−2^sec^−1^ light), were used for the experiment. The 100 µL elicitor master mix for each sample contained 50 nM flg22, 100 μM luminol (Sigma) and 20 μg/mL HRP (Sigma). ROS was elicited with 50 nM Pto_flg22, and the luminescence was measured over 60 min using a Microplate luminescence reader (Varioskan flash, Thermo Scientific, USA). The flg22 peptide was purchased from Abclonal (China). The peptide sequences are Pto_flg22: TRLSSGLKINSAKDDAAGLQIA.

### MAP kinase assay

The MAPK activation assays were performed as previously described [69]. The seedlings were grown under long-day condition (22 °C, 16 h light/8 h dark, 120 µmol photons m^−2^ s^−1^ light). Seven-day-old seedlings were transferred from solid 1/2 MS plates to 12-well plates with 1.5 mL liquid 1/2 MS in each well. Fourteen-day-old *Arabidopsis* plants were treated with 100 nM Pto_flg22 and samples were collected at different time points as indicated in the figure. The samples were ground with tissue-lyzer under frozen liquid nitrogen. To each sample, 100 µL protein extraction buffer (100 mM Tris-HCl pH 7.5, 150 mM NaCl, 5 mM EDTA, 10% glycerol, 1 mM Phenylmethylsulfonyl fluoride (PMSF), 1% protease inhibitor, 5 mM dithiothreitol (DTT), 10 mM sodium fluoride, 10 mM sodium molybdate, 2 mM Na_3_VO_4_, 0.1% NP-40) were added. The samples were then vortexed and thawed on ice. After that, the samples were centrifuged at 16,000 g for 10 min at 4 °C. Twenty µL of 5× SDS loading buffer was added into 80 µL supernatant and denatured at 70 °C for 10 min. The samples were then cooled at room temperature and centrifuged at 16,000  *g* for 1 min. Protein samples were separated on 10% SDS-PAGE gels, and the western blots were probed with anti pMAPK antibodies. The first antibody was Phospho-p44/42 (Cell Signaling Technology, Cat No. 4370L; 1:2000 dilution). The second antibody was goat anti-rabbit HRP (Sigma A0545; 1:5000 dilution).

### *Pst*. DC3000 and flg22 treatments

The *Pst*. DC3000 treatments were performed following Yu et al. [70]. Four-week-old *Arabidopsis* plants grown in short-day condition (22 °C, 10 h light /14 h dark, 120 µmol photons m^−2^sec^−1^ light) were used for the infiltration assay. The DC3000 strains was suspended in water to a concentration of OD = 0.0002 for infiltration. Three leaves were infected for each plant. The *Pst*. DC3000 solution was infiltrated into leaves on the abaxial side with a syringe without needle. After 3 days, the leaf discs were collected by biopsy punch with a diameter of 7 mm. Three leaf discs were collected for each plant and kept in one tube with 500 µL of sterile water and three metal beads inside. Then the samples were ground by tissue-lyzer at least 30 s with a frequency of 25 cycles per second until completely homogenized. A series of at least 1/10 dilution of the solution was made in 96-well plates. The 20 µL of each dilution was dropped on LB plates with 25 ng/µL kanamycin and 25 ng/µL rifampin. The plates were placed at 28 °C for 36 h before the colonies were counted.

For flg22-induced PTI marker gene *FRK1* expression analysis, *Arabidopsis* seedlings grown on 1/2 MS plates for 6 days were dipped in 100 nM flg22 solution. Seedlings dipped in water were used as the control. Samples were collected at different time points as indicated in the figure legends. Each biological replicate included 15 seedlings.

## Supplementary information

Supplementary Figures

Dataset S1

Dataset S2

Dataset S3

Table S1

Table S2

Table S3

## Data Availability

The raw RNA-seq data is available in the NCBI GEO with the accession number GSE173239; The 16S rRNA gene sequencing data is available in the NCBI SRA under BioProject PRJNA723970.
