# Supplementary material for: Dysfunction of histone demethylase IBM1 in Arabidopsis causes autoimmunity and reshapes the root microbiome
Source: ISME J. 2022 Jul 30;16(11):2513–24. doi: 10.1038/s41396-022-01297-6 (PMC9561531; doi:10.1038/s41396-022-01297-6)
Supplement: Supplementary file 1 — Supplementary Figures [file 41396_2022_1297_MOESM1_ESM.pdf]

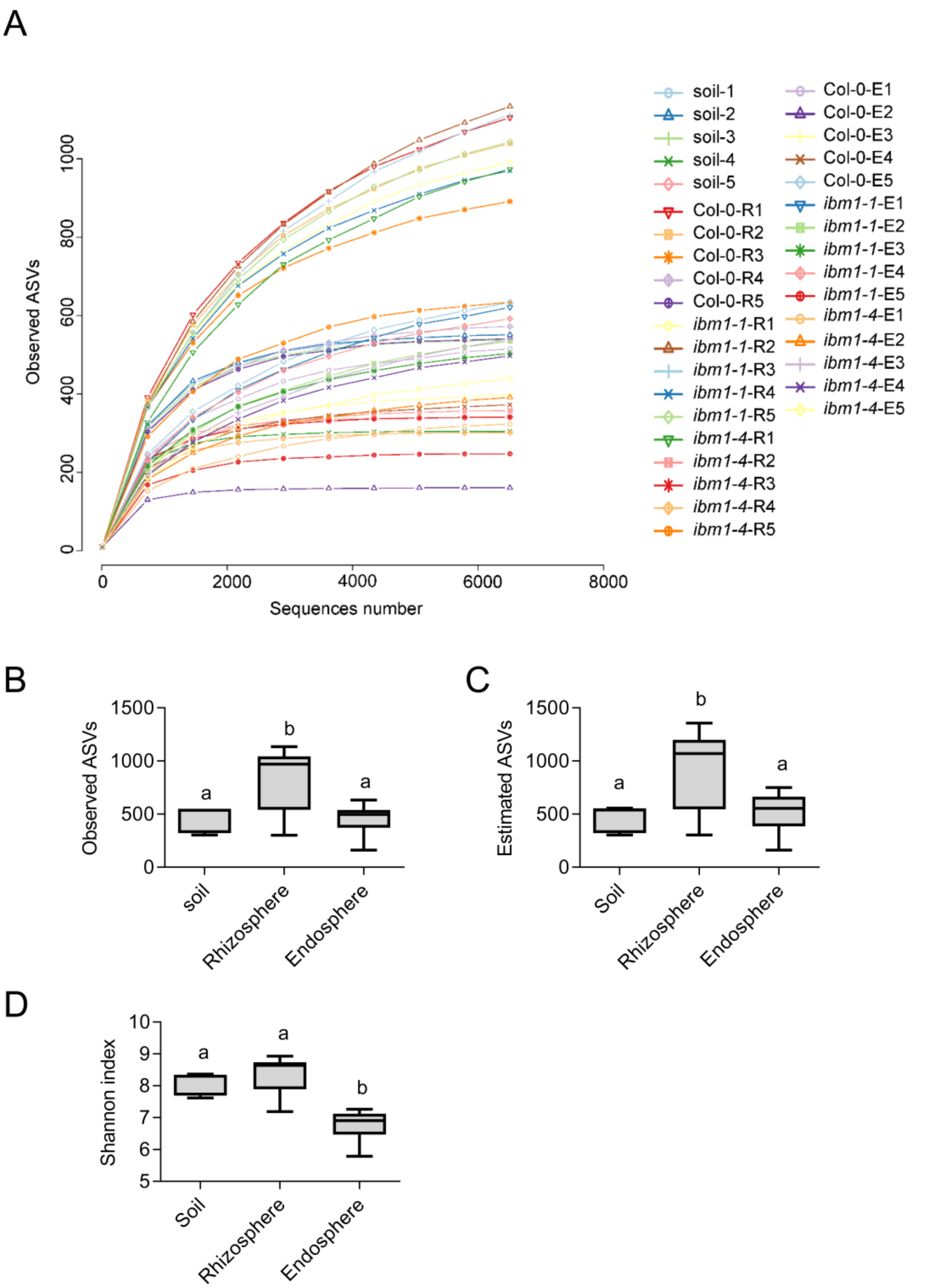

Figure S1. Bacteria richness in different compartments reflects the selectivity of plants on root-associated microbes (Related to Figure 1).

[A] Rarefaction curves of each sample. **[B]** Numbers of observed ASVs in the different compartments. **[C]** Numbers of estimated ASVs based on the Chao1 estimator. **[D]** Shannon index of the microbe diversity. The analysis was based on the table of normalized absolute abundance of ASVs. Letters denote statistical significance (*p* < 0.05, Turkey).

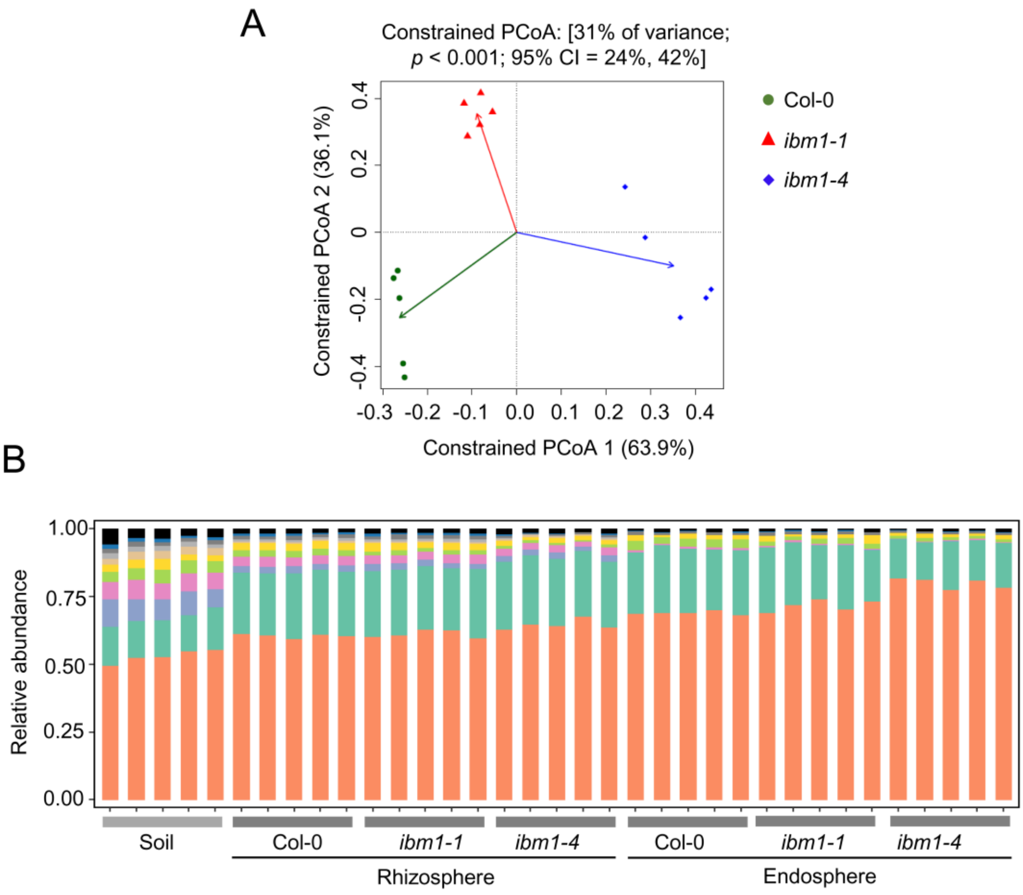

	Rhizosphere *ibm1-1*	Rhizosphere *ibm1-4*	Endosphere *ibm1-1*	Endosphere *ibm1-4*
Proteobacteria		* (red)		* (red)
Bacteroidetes		* (red)		* (green)
Acidobacteriota				
Verrucomicrobiota	* (green)	* (green)		
Myxococcota	* (green)	* (green)	* (green)	* (green)
Actinobacteriota		* (green)		
Nitrospirota				
Gemmatimonadota		* (green)		
Bdellovibrionota		* (green)		
Planctomycetota		* (green)		
Others				

* (red) Enriched in *ibm1* (FDR< 0.05)
* (green) Decreased in *ibm1* (FDR< 0.05)

Figure S2. IBM1 dysfunction alters plant-associated microbiome (Related to Figure 1).

[A] The PCoA analysis of all ASVs separates the *ibm1* mutants from the wild type plants (Col-0) within the rhizosphere compartment. **[B]** Relative abundance (RA) of the top 10 bacteria phyla detected within the bulk soil (Soil), rhizosphere, and endosphere compartments. Statistical significance (FDR < 0.05) of phylum RA between the mutant and the wild type plants is indicated by asterisks in the table.

A

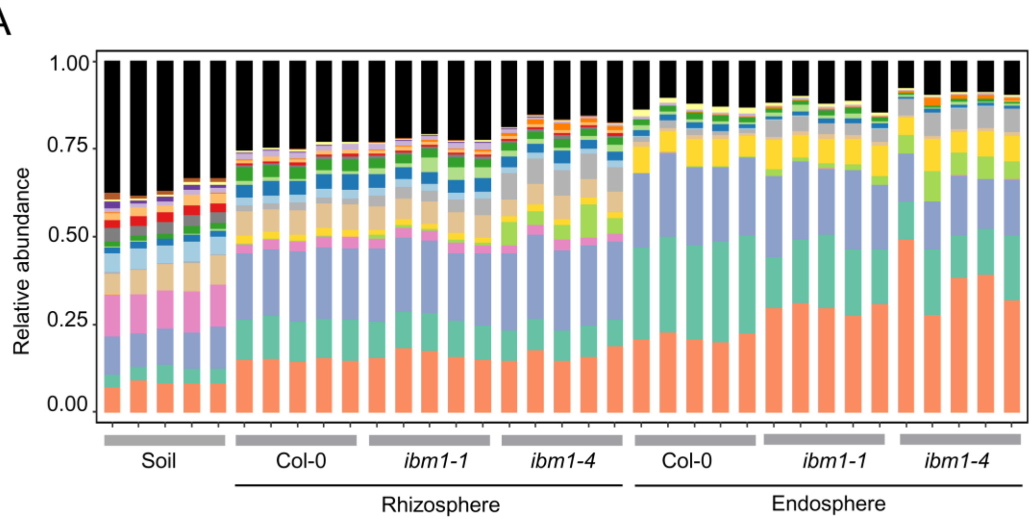

B

	Rhizosphere		Endosphere	
	ibm1-1	*ibm1-4*	*ibm1-1*	*ibm1-4*
Comamonadaceae			* (enriched)	* (enriched)
Oxalobacteraceae	* (decreased)	* (decreased)	* (decreased)	* (decreased)
Flavobacteriaceae		* (enriched)		* (decreased)
Nitrosomonadaceae				
Aeromonadaceae	* (enriched)	* (enriched)	* (enriched)	* (enriched)
Rhizobiaceae	* (decreased)			
Sphingomonadaceae	* (decreased)	* (decreased)		
Pseudomonadaceae	* (enriched)	* (enriched)	* (enriched)	* (enriched)
Pedosphaeraceae	* (decreased)			
Xanthomonadaceae		* (decreased)		* (decreased)
Cellvibrionaceae				
Caulobacteraceae	* (decreased)	* (decreased)		* (decreased)
TRA3-20				
Nitrospiraceae				
SC-I-84				
Enterobacteriaceae	* (enriched)	* (enriched)	* (enriched)	* (enriched)
Microscillaceae		* (decreased)		* (decreased)
Vicinamibacteraceae				
Sandaracinaceae	* (decreased)	* (decreased)	* (decreased)	* (decreased)
Subgroup_5				
Other classify				

* Enriched in *ibm1* (FDR< 0.05)
* Decreased in *ibm1* (FDR< 0.05)

Figure S3. Taxonomic structure of ASVs (Amplicon Sequence Variants) reveals the impacts of IBM1 dysfunction on root microbiome at family level (Related to Figure 1).

[A] Relative abundance (RA) of the top 20 bacteria families detected within the bulk soil (Soil), rhizosphere, and endosphere compartments. **[B]** A table summarizing statistical significance (FDR < 0.05 as indicated by asterisks) between the mutant and the wild type plants of family RA as showed in panel A.

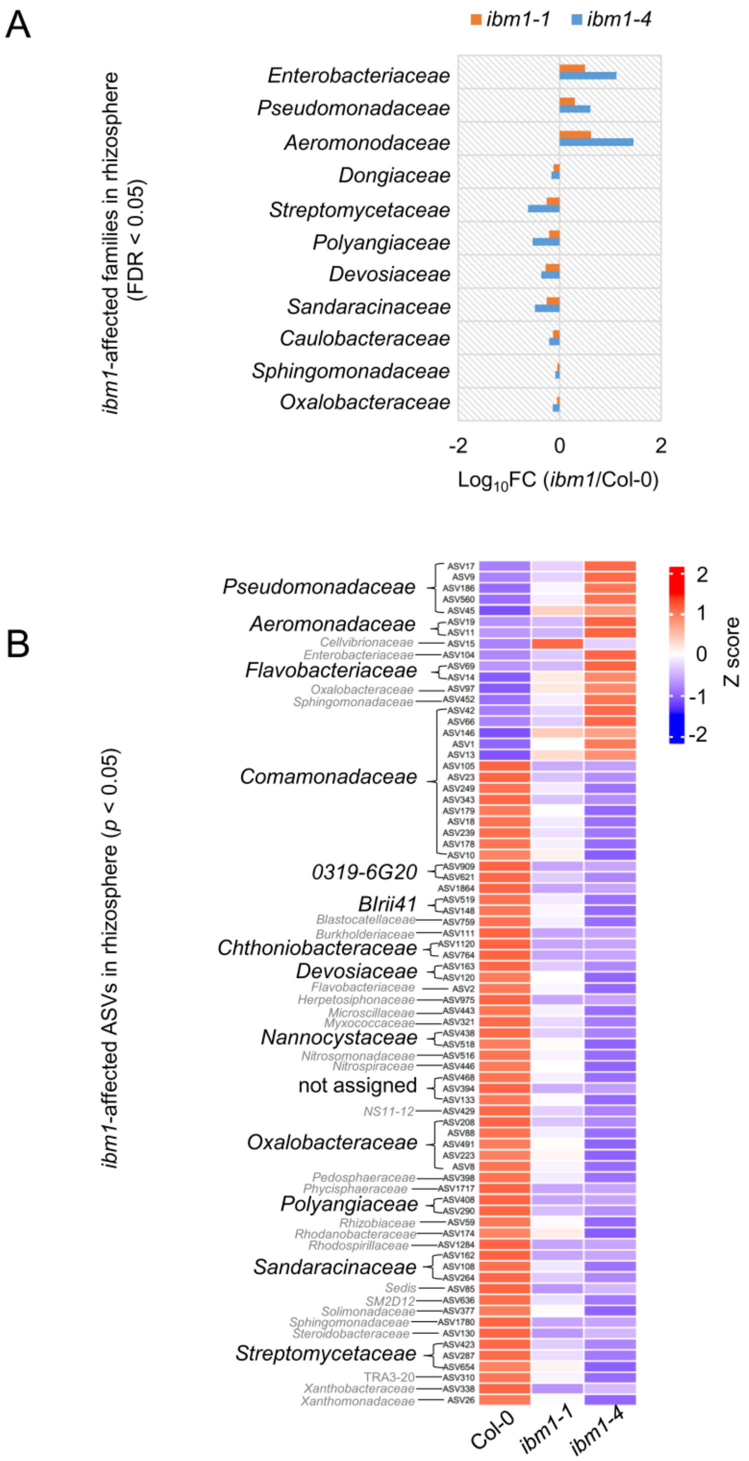

Figure S4. IBM1 dysfunction reshapes Arabidopsis rhizosphere microbiome (Related to Figure 1). **[A]** The *ibm1* mutations resulted in altered (FDR < 0.05 for both the *ibm1-1* vs Col-0 and the *ibm1-4* vs Col-0) RA in 11 bacteria families within the rhizosphere microbiome. The Log_{10} fold changes of mutant vs wild type are shown as horizontal bars. **[B]** The *ibm1* mutations resulted in altered (p < 0.05 for both the *ibm1-1* vs Col-0 and the *ibm1-4* vs Col-0) RA in 76 rhizosphere ASVs in the mutants compared to the wild type. The left notes indicate the taxonomy of each ASV at family level.

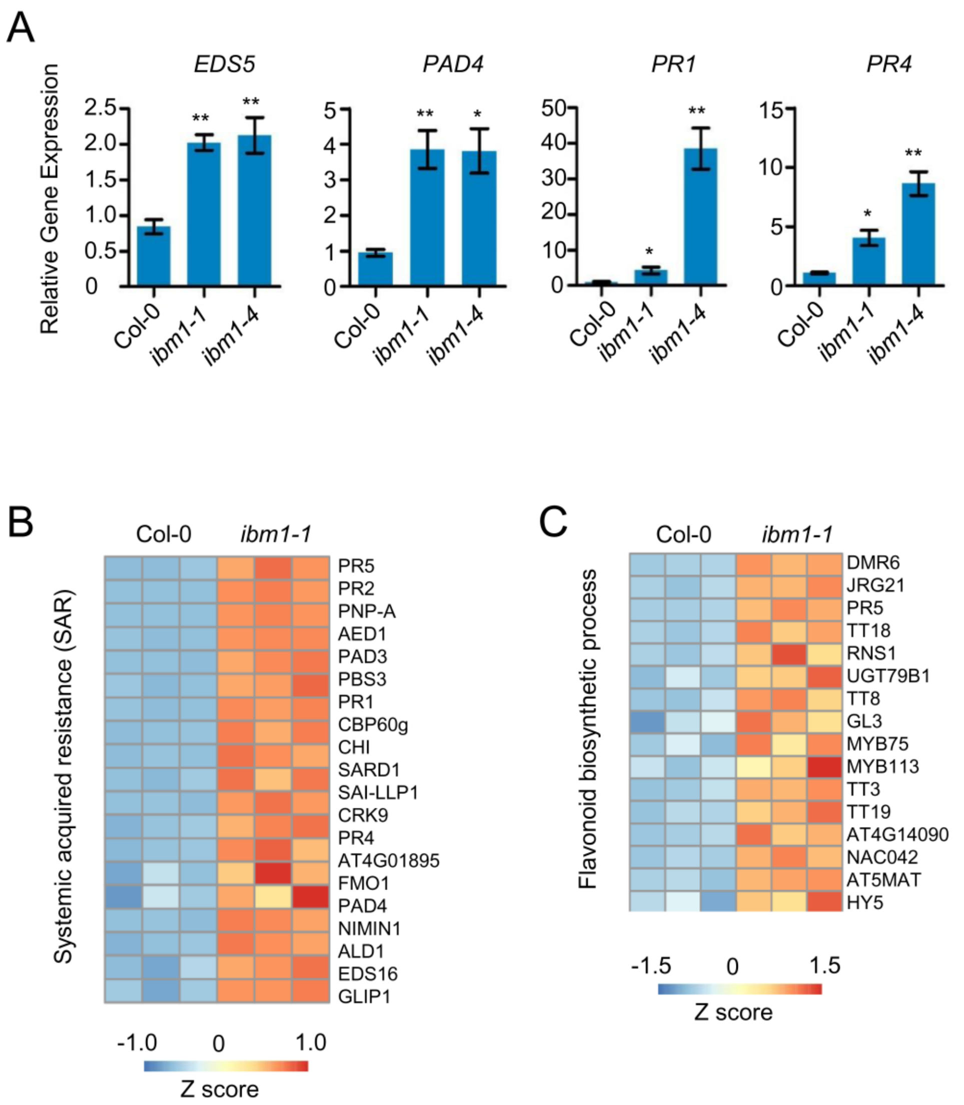

Figure S5. IBM1 dysfunction activates defense-related responses in soil-grown Arabidopsis (Related to Figure 2).

[A] Gene expression levels of *EDS5*, *PAD4*, *PR1* and *PR5* in 2-week-old Col-0, *ibm1-1* and *ibm1-4* plants. Results of RT-qPCR are shown. Mean ± SE, n = 3 biological replicates. * and ** indicate $p < 0.05$ and $p < 0.01$, respectively (Student's t-test). **[B]** A heatmap of DEGs (FC > 2, FDR < 0.05) involved in systemic acquired resistance (SAR). **[C]** A heatmap of DEGs involved in the flavonoid biosynthesis process.

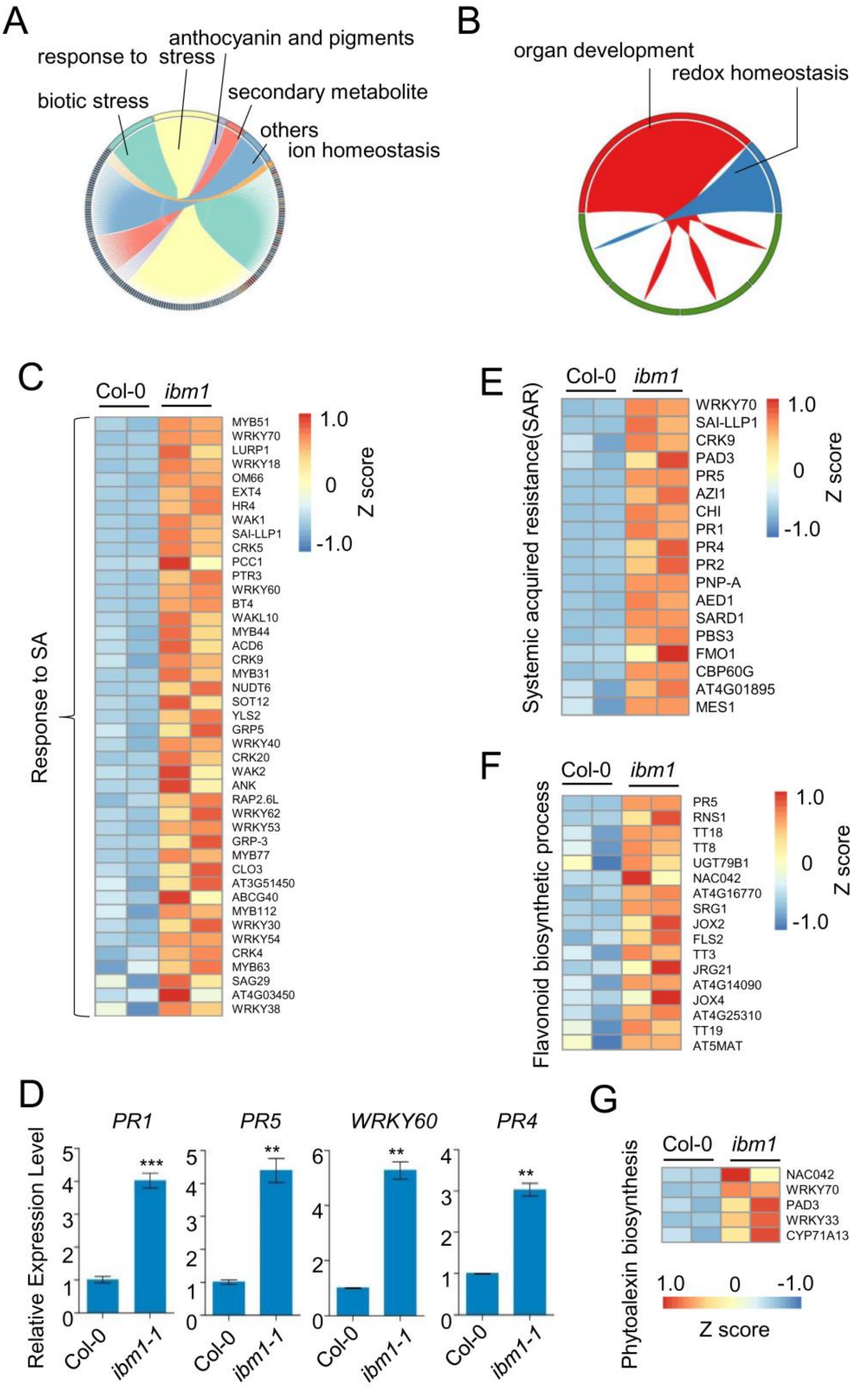

Figure S6. IBM1 dysfunction causes autoimmunity in Arabidopsis grown in sterile medium (Related to Figure 2).

The differentially expressed genes (DEGs; fold change ≥ 2, FDR ≤ 0.05) that were up-regulated **[A]** and down-regulated **[B]** in *ibm1-1* compared to Col-0 were subject to the Gene Ontology (GO) analysis. The chord diagrams show the GO terms that link to their sub-classifications. The sub-

classifications are labeled with GO ID that can be queried together with their corresponding DEGs in Dataset S2 (Sheet 9 and 11). **[C]** A heatmap of DEGs involved in SA signaling or biosynthesis. **[D]** Gene expression levels of *PR1, PR4, PR5,* and *WRKY60* in 7-day-old Col-0 and *ibm1-1* plants grown in sterile half-strength MS medium. Results of RT-qPCR are shown. Mean ± SE, n = 3 technical replicates. Two biological replicates were analyzed with similar results. ** indicate p < 0.01 (Student's t-test). **[E]** A heatmap of DEGs involved in systemic acquired resistance (SAR). **[F]** A heatmap of DEGs involved in the flavonoid biosynthesis process. **[G]** A heatmap of DEGs involved in phytoalexin biosynthesis.

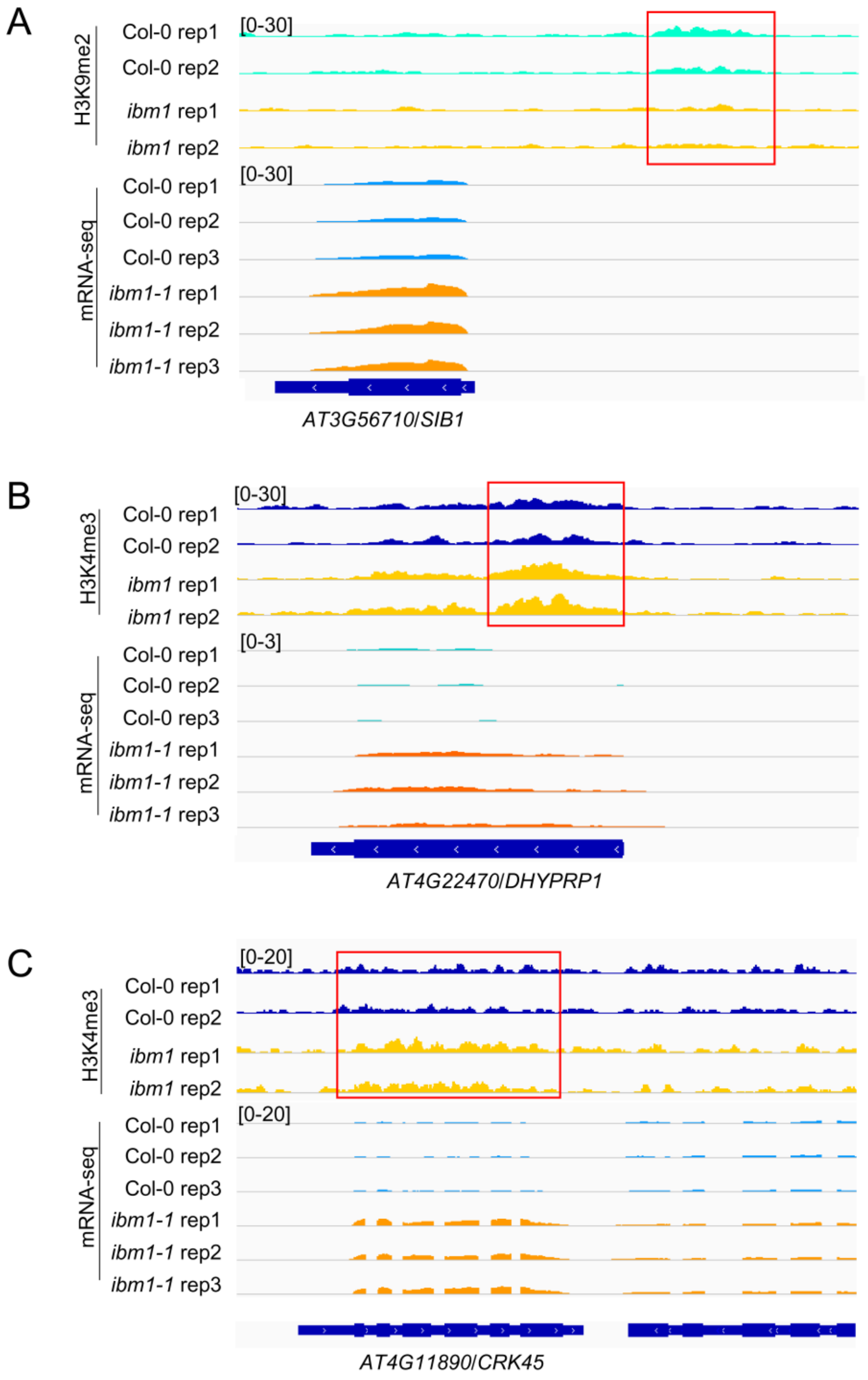

Figure S7. IBM1 dysfunction results in histone modifications permissive for transcription of some important defense genes (Related to Figure 3).

[A] IBM1 dysfunction decreases H3K9me2 level at the gene promoter region of *SIGMA FACTOR BINDING PROTEIN1* (*SIB1*) and increases the mRNA level of *SIB1*. Snapshots from ChIP-seq and RNA-seq are shown. The original ChIP-seq data were downloaded from DDBJ (DRA005154) as generated previously (24). The red box indicates the region with altered H3K9me2 levels. **[B]** IBM1 dysfunction increases H3K4me3 level at the gene body region of *DHYPRP1* and increases the mRNA level of *DHYPRP1*. **[C]** IBM1 dysfunction increases H3K4me3 level at the gene body region of *CRK45* and increases the mRNA level of *CRK45*.

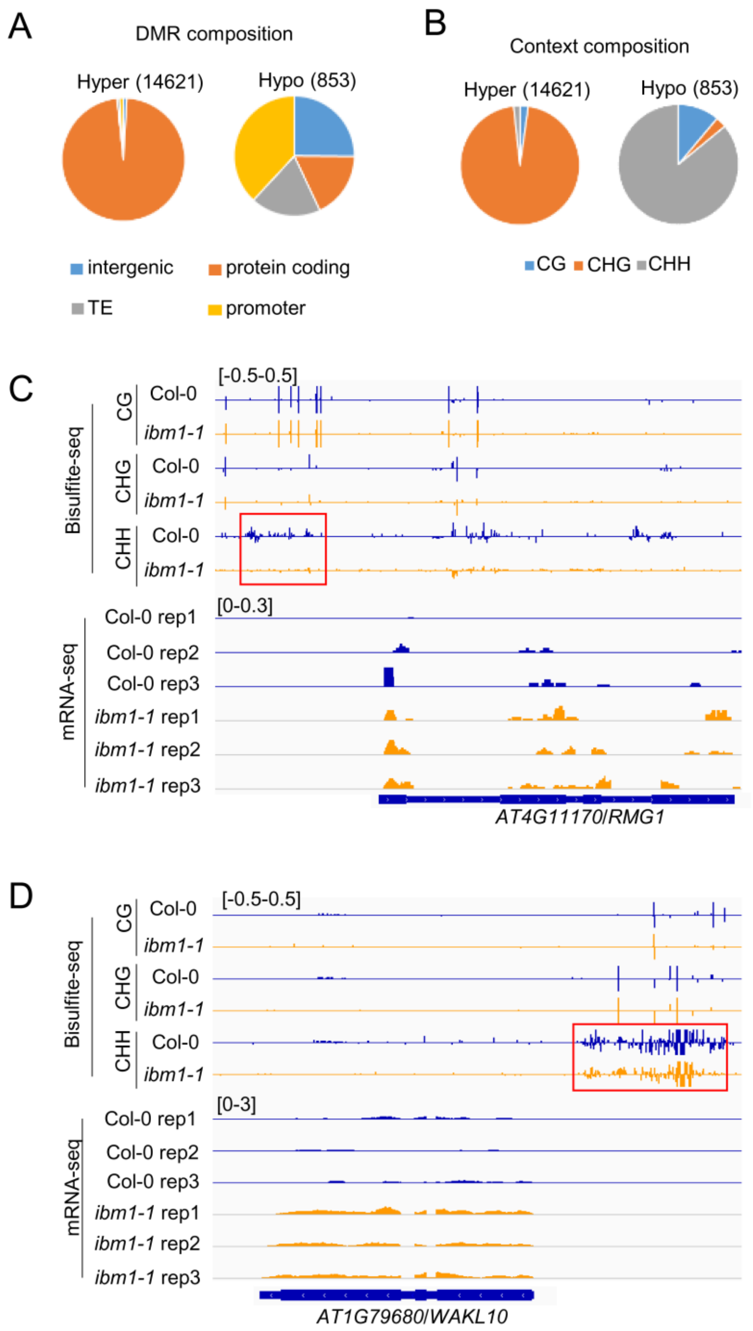

Figure S8. IBM1 dysfunction decreases DNA methylation levels in the promoter regions of some important defense genes (Related to Figure 3).

[A] The composition of the hyper methylated and hypo methylated DMRs (differentially methylated regions) caused by IBM1 dysfunction. **[B]** The composition of differentially methylated cytosines in the CG, CHG, and CHH contexts. **[C]** IBM1 dysfunction decreases DNA methylation level at the promoter region of *RMG1* and increases the mRNA level of *RMG1*. Snapshots from whole genome bisulfite sequencing and RNA-seq are shown. The red box indicates the gene promoter region with altered CHH methylation levels. **[D]** IBM1 dysfunction decreases DNA methylation level at the promoter region of *WAKL10* and increases the mRNA level of *WAKL10*.

A

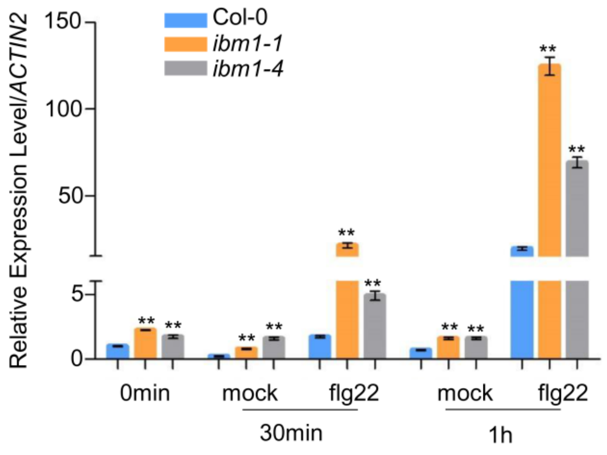

B

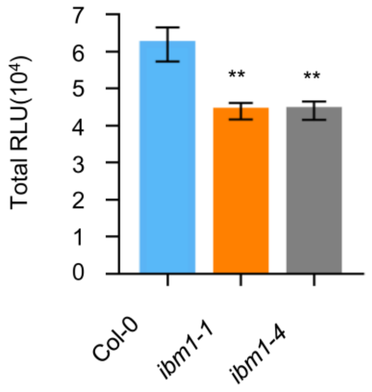

Figure S9. IBM1 dysfunction alters flg22-induced plant immune responses (Related to Figure 4). **[A]** Gene expression levels of the PTI marker gene *FRK1*. Seven-day-old *ibm1* mutants and wild type (Col-0) plants were dip-inoculated with 100 nM flg22 and harvested at the indicated time points. Results of RT-qPCR are shown. Mean ± SE, n = 3 technical replicates. Two biological replicates were analyzed with similar results. ** indicate $p < 0.01$ (Student's t-test). **[B]** Measurements of ROS burst induced by flg22. Leaf discs from four-week-old plants elicited with 50 nM flg22. Mean ± SE, n = 3 biological replicates, each consisting of 16 leaf discs.

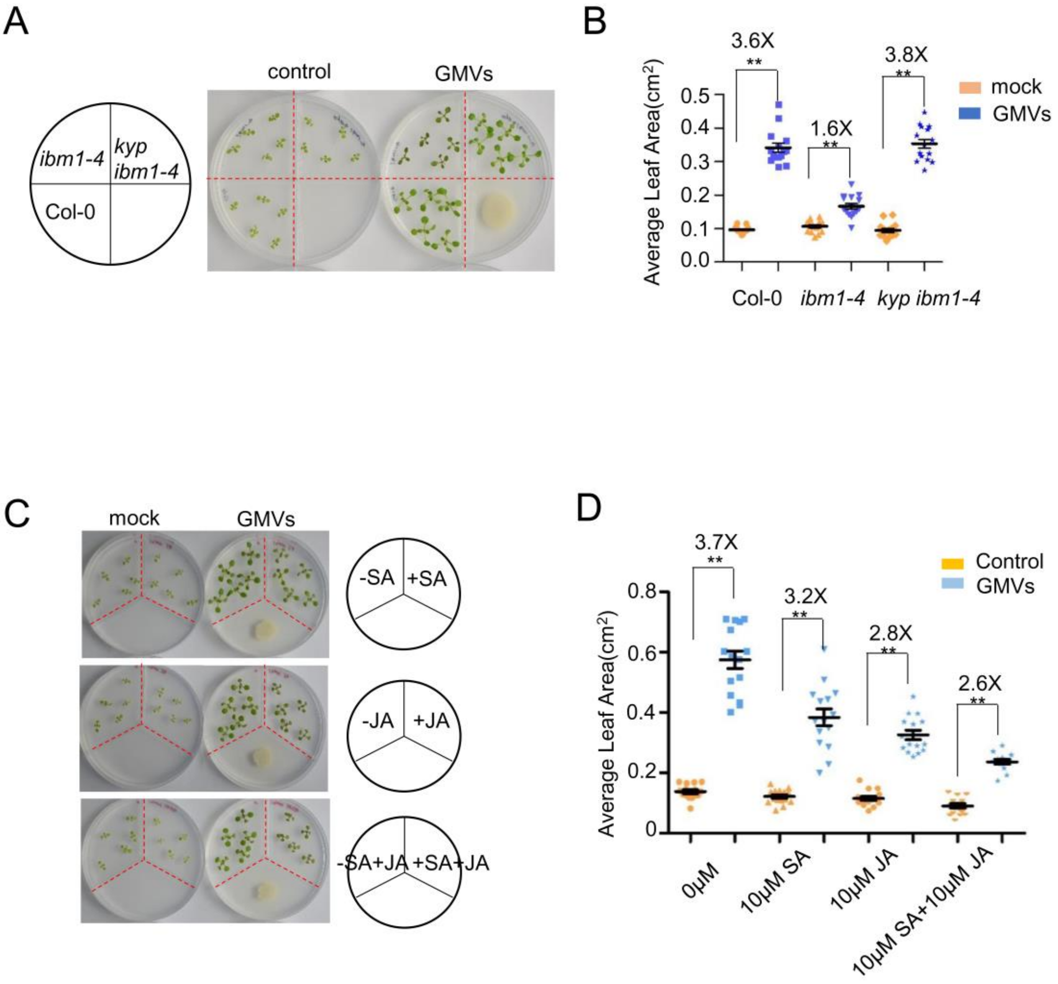

Figure S10. Dysfunction of IBM1 and activation of defense impair plant growth-promotion triggered by GMVs (Related to Figure 4).

[A] IBM1 dysfunction impairs plant growth-promotion triggered by GB03-produced microbial volatiles (GMVs); the impairment can be rescued by a second mutation of *kyp* in the *ibm1* mutant. Images were taken at 7 days after treatment (DAT). Red-dotted lines indicate inner plastic partitions that divide the plate into four parts. **[B]** Quantification of total leaf area per seedling (TLA) of the plants at 7 DAT. Mean ± SE, n = 15. All fold changes are associated with statistical significance of $p < 0.01$ (Student's t-test). **[C]** Exogenous application of SA and/or JA mimicked *ibm1* mutations in impairing GMV-triggered plant growth-promotion. Images were taken at 7 days after treatment (DAT). **[D]** Quantification of TLA of the SA/JA-treated plants at 7 DAT. Mean ± SE, n = 15. All fold changes are associated with statistical significance of $p < 0.01$ (Student's t-test).

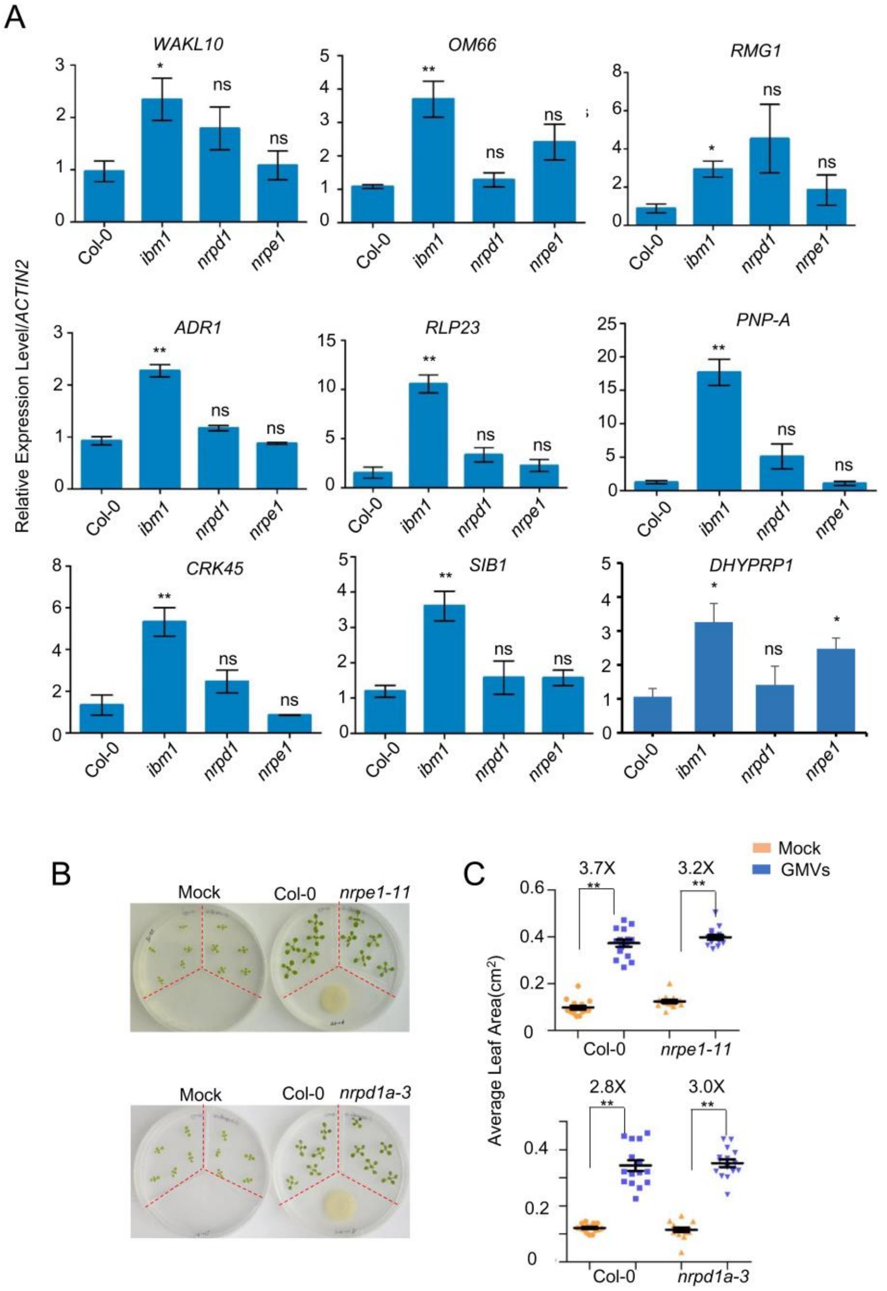

Figure S11. IBM1 has stronger impacts on plant-microbe interactions than the RNA-directed DNA methylation (RdDM) pathway (Related to Figure 4).

[A] IBM1 dysfunction (*ibm1-1*), but not defective RdDM (*nrpd1-3* and *nrpe1-11*), induces gene expression of all the examined defense regulators. Results of RT-qPCR are shown. Mean ± SE, n = 3 biological replicates. * and ** indicate $p < 0.05$ and $p < 0.01$, respectively (Student's t-test). **[B]** The RdDM mutants *nrpd1-3* and *nrpe1-11* showed similar plant growth-promotion as wild type plants (Col-0) in response to GMVs. Images were taken at 6 days after treatment (DAT). Red-dotted lines indicate inner plastic partitions that divide the plate into three parts. **[C]** Quantification of total leaf area per seedling (TLA) of the plants at 6 DAT. Mean ± SE, n = 15 seedlings in three biological replicates. All fold changes are associated with statistical significance of $p < 0.01$ (Student's t-test).
